# Uncovering the mechanisms underlying the efficacy of probiotic strains in mitigating food allergies: an emphasis on gut microbiota and indoleacrylic acid

**DOI:** 10.3389/fnut.2024.1523842

**Published:** 2024-12-12

**Authors:** Zhangming Pei, Li Qian, Taolin Miao, Hongchao Wang, Wenwei Lu, Yuqing Chen, Qianger Zhuang

**Affiliations:** ^1^State Key Laboratory of Food Science and Resources, Jiangnan University, Wuxi, China; ^2^School of Food Science and Technology, Jiangnan University, Wuxi, China; ^3^Children's ENT Department, Affiliated Children’s Hospital of Jiangnan University (Wuxi Children’s Hospital), Wuxi, China; ^4^National Engineering Research Center for Functional Food, Jiangnan University, Wuxi, China; ^5^Children's ENT Department, Affiliated Women’s Hospital of Jiangnan University (Wuxi Maternal and Child Healthcare Hospital), Wuxi, China

**Keywords:** food allergy, probiotic, antibiotic cocktail, gut microbiota, indoleacrylic acid

## Abstract

Food allergies manifest as systemic or digestive allergic responses induced by food allergens, and their progression has been demonstrated to be intimately associated with the host’s gut microbiota. Our preceding investigation has revealed that the probiotic strains *Lactiplantibacillus plantarum* CCFM1189 and *Limosilactobacillus reuteri* CCFM1190 possess the capability to mitigate the symptoms of food allergy in mice. However, the underlying mechanisms and material foundations through which these probiotic strains exert their effects remain enigmatic. Here, we initially compared the ameliorative effects of these two probiotic strains on food allergy mice subjected to antibiotic cocktail (ABX) treatment. It is indicated that ABX treatment was ineffective in alleviating weight loss, diarrhea, and allergic symptoms in mice, and it also inhibited the reduction of histamine and T helper cell 2 (Th2) cytokines mediated by effective strains, suggesting that effective strains must operate through the gut microbiota. Then, building upon the outcomes of prior non-targeted metabolomics studies, by quantifying the content of indoleacrylic acid (IA) in single-strain fermentation of probiotic strains and mouse feces, it was ascertained that effective strains do not synthesize IA themselves but can augment the concentration of IA in the gut by modulating the gut microbiota. Ultimately, we discovered that direct intervention with IA could mitigate diarrhea, allergic symptoms, and intestinal damage by modulating immunoglobulin E (IgE) levels, histamine, Th2 cytokines, and tight junction proteins, thereby corroborating that IA is a pivotal metabolite for the alleviation of food allergies. These observations underscore the significance of gut microbiota and metabolites like IA in the management of food allergies and hold potential implications for the development of novel therapeutic strategies.

## Introduction

Food allergies are immune system disorders triggered by protein antigens in food, resulting in a range of clinical symptoms that can affect the gastrointestinal tract, respiratory system, skin, and central nervous system (1). Currently, there is no complete cure for food allergies. The most common approach to managing these conditions is the strict avoidance of allergenic foods to prevent reactions (2). However, due to insufficient regulation of food labeling, identifying potential allergenic components in food products remains challenging. Consequently, there is an urgent need to explore new methods for treating food allergies. The gut microbiota has been acknowledged as a critical factor in the etiology and regulation of allergic diseases (3, 4). The gut microbiota engages in direct interactions with the intestinal mucosal surface, thereby modulating the host’s immune response via diverse mechanisms, such as the regulation of immune cell populations (5), the synthesis of antimicrobial peptides (6), and the maintenance of intestinal barrier integrity (7). Researchers hypothesize that inadequate exposure to specific beneficial microbes during early life may contribute to the escalating prevalence of allergic conditions (8), underscoring the microbiota’s indispensable role in the maturation and function of the immune system. Based on this, probiotic therapy is regarded as a promising strategy to mitigate allergic diseases by modulating the gut microbiota (9).

Recent studies have demonstrated that probiotics exert a significant impact on reducing specific immunoglobulin E (sIgE) concentrations, balancing the T helper cell 1 (Th1)/T helper cell 2 (Th2) immune axis, fine-tuning dendritic cell activity, suppressing mast cell activation, and augmenting intestinal barrier integrity in subjects afflicted with food allergies (10–12). These multifaceted effects are hypothesized to ameliorate food allergy manifestations by rectifying intestinal immune dysregulation, optimizing gut microbiota diversity and configuration, and harmonizing gut metabolic processes. However, there is a paucity of analysis on the mechanism and material basis of probiotics in alleviating food allergies, and research on the relationship between food allergies and intestinal metabolism remains limited. By introducing beneficial bacteria into the gut, probiotics are capable of altering the composition and metabolic activity of the resident microbiota (13, 14). Intestinal small molecule metabolites, which are byproducts of microbial metabolism, exert significant effects on the host’s immune response and intestinal barrier function. These metabolites encompass short-chain fatty acids, bile acids, and tryptophan-indole derivatives, all of which are known for their anti-inflammatory properties and ability to strengthen the intestinal barrier (15). Thus, probiotic strains that foster the growth of bacteria capable of producing such beneficial metabolites may aid in regulating intestinal immune homeostasis and enhancing barrier function, potentially mitigating symptoms of food allergy in mice and possibly in humans as well.

In a prior study, we demonstrated that *Lactiplantibacillus plantarum* CCFM1189 and *Limosilactobacillus reuteri* CCFM1190 have the potential to mitigate food allergy symptoms in ovalbumin (OVA)-induced mice (16). Based on the findings from untargeted metabolomics analysis, it is speculated that the efficacy of these strains may be mediated by the Gut microbiota specific small molecule metabolite, indoleacrylic acid (IA). Therefore, in this study, we initially utilize an antibiotic cocktail (ABX)-treated mouse model to examine whether the two efficacious probiotic strains augment the fecal concentration of IA in mice by modulating the gut microbiota, thus restoring the Th1/Th2 immune balance in food-allergic mice and alleviating the pathological manifestations associated with food allergy. Furthermore, we directly administer IA to mice to validate its efficacy, thereby confirming whether IA is the active component responsible for the alleviation of food allergy.

## Materials and methods

### Cultivation of probiotic strains used in this study

The probiotic strains, *L. plantarum* CCFM1189 and *L. reuteri* CCFM1190, were obtained from the Culture Collection of Food Microorganisms at Jiangnan University (Wuxi, China). CCFM1189 was incubated under anaerobic conditions in de Man-Rogosa-Sharpe (MRS) liquid medium at 37°C for a duration of 16–24 h. CCFM1190 was cultivated in MRS liquid medium supplemented with 0.5 g·L^−1^ L-cysteine hydrochloride within an anaerobic chamber at 37°C for a period of 24–48 h. After strain amplification, centrifuge at 4°C and 8,000 × *g* for 10 min, discard the supernatant, wash twice with 0.9% sterile saline, resuspend the bacterial slurry in 5 mL of sucrose solution, count, and store in a − 80°C freezer. Before gavage, centrifuge at 4°C and 8,000 × *g* for 10 min, dilute the bacterial suspension to 5 × 10^9^ CFU·mL^−1^ with 0.9% sterile saline based on the counting results, and set aside.

### Preparation of ABX solution and IA solution

The dosage regimen for the ABX solution in this study was adapted from Scott et al. (17): Ampicillin (9 mg·kg^−1^), metronidazole (9 mg·kg^−1^), neomycin sulfate (9 mg·kg^−1^), and vancomycin (4.5 mg·kg^−1^) were administered via intragastric route at a volume of 0.2 mL per day, commencing from the adaptation phase and continuing until the 30th day, which precedes the initiation of the stimulation protocol.

The IA was solubilized using a Dimethyl sulfoxide (DMSO) solution (18). The composition of the DMSO solution included DMSO, polyethylene glycol 300 (PEG300), tween-80, and saline solution in the ratio of 2:8:1:9, respectively. The IA was dissolved in this DMSO solution. The low-dose IA group (IA1) received 0.2 mL of the low-dose IA solution (4.8 mg·mL^−1^), whereas the high-dose IA group (IA2) was administered 0.2 mL of the high-dose IA solution (48 mg·mL^−1^).

### Animals and experimental protocol

**Figure 1:**
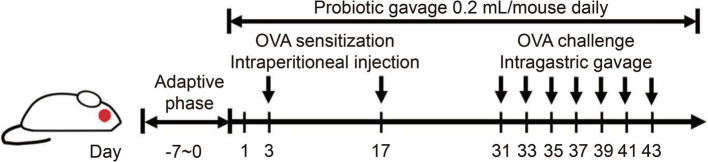
The procedure for modeling OVA-induced food allergy and intervention cycle in mice.

The mice used in this experiment were specific pathogen free (SPF) 4-week-old female BALB/c mice, which were purchased from Charles River Laboratory Animal Technology Co., Ltd. (Zhejiang, China), and raised in the barrier facilities of the Experimental Animal Center at Jiangnan University. The initial body weights of the mice ranged from 13 to 16 g. A one-week acclimatization period was observed prior to the commencement of the experiment. The environmental conditions for feeding, the dietary regimen, the establishment of the food allergy model, and the protocols for execution and sampling were all in accordance with the methodologies detailed in our previous study (16), the schematic diagram is shown in Figure 1.

The mice were randomly allocated into 10 distinct groups: control, model, ABX, ABX + CCFM1189, ABX + CCFM1190, CCFM1189, CCFM1190, DMSO, IA1, and IA2. Each group comprised 6–7 mice. Mice in the ABX + CCFM1189, ABX + CCFM1190, CCFM1189, and CCFM1190 groups received a daily gavage of 0.2 mL (5 × 10^9^ CFU·mL^−1^) of probiotic suspension from day 0 to day 43. Conversely, mice in the control and model groups were administered an equivalent volume of sterile saline solution. For the ABX-treated groups (ABX, ABX + CCFM1189, ABX + CCFM1190), each mouse received a daily gavage of 0.2 mL of ABX solution from the adaptation period until the 30th day (the day preceding the initiation of stimulation), with the ABX being dissolved in saline solution. The DMSO group received intragastric administration of 0.2 mL of DMSO solution from day 0 to day 43. For the IA-treated groups, the IA1 group received 0.2 mL of low-dose IA solution (4.8 mg·mL^−1^) per mouse from day 0 to day 43, while the IA2 group received 0.2 mL of high-dose IA solution (48 mg·mL^−1^) per mouse over the same period. The mice were weighed at the same time each week from the adaptive period, with their weights expressed as percentages (weight% = current weight / initial weight (day 0) × 100%).

This investigation was conducted in full compliance with the NIH Guidelines for the Care and Use of Laboratory Animals (NIH Publication No. 85-23 Rev. 985) and received formal approval from the Laboratory Animal Ethics Committee of Jiangnan University (Wuxi, Jiangsu, China; approval numbers JN. No. 20210915b1121201[300] and JN. No. 20211115b0581228[434]).

### Assessment of food allergy symptom score and diarrhea index in mice

The calculation method of food allergy symptom score in mice detailed in our previous study (16). Feces of mice were collected for a period of time, and the diarrhea index was calculated (diarrhea index = the number of soft stools / the total amount of feces).

### Determination of OVA-sIgE, interleukin (IL)-4, IL-5, IL-13, and histamine (HIS) contents

Serum and jejunal tissue samples were procured and subjected to analysis using mouse-specific OVA-sIgE, IL-4, IL-5, IL-13, and HIS enzyme-linked immunosorbent assay (ELISA) kits (Nanjing Senbeijia Biological Technology Co., Ltd., Nanjing, China), in accordance with the manufacturer’s guidelines. HIS, a biogenic amine present in the human body, is primarily recognized for its function as a mediator in allergic responses; however, it also serves as a crucial signaling molecule within the nervous system, gastrointestinal tract, integumentary system, and immune system. The absorbance was quantified at a wavelength of 450 nm utilizing a microplate reader (Thermo Fisher Scientific, Waltham, MA, United States).

### Immunohistochemical analysis of tight junction proteins zonula occludens 1 (ZO-1) and occludin in jejunal tissue of mice

The jejunal tissues were preserved in a 4% paraformaldehyde solution. Subsequently, the tissues were processed for immunohistochemical sectioning, encompassing steps such as repair, dehydration, transparency, waxing, embedding, sectioning, dewaxing, antigen retrieval, sealing, incubation with primary and secondary antibodies, Diaminobenzidine coloration, termination of coloration, and redyeing and dewatering. The sections were digitized and examined at 400× magnification using a digital slide scanner (3DHISTECH Ltd., Hungary). The positive staining area and total area were quantified using Image J 1.8.0 (National Institutes of Health, United States).

### *In vitro* fermentation assay of probiotic strains

The CCFM1189 and CCFM1190 strains were activated using MRS liquid medium over two generations. The second generation was harvested at the logarithmic phase, centrifuged at 4000 × *g* for 10 min, and the supernatant was subsequently discarded. An equivalent volume of the pre-reduced isotonic potassium phosphate buffer (PPS; 10 mM MgSO_4_, 21.46 mM KH_2_PO_4_, 18.54 mM K_2_HPO_4,_ pH 7.0) was prepared for the purpose of cleansing bacterial cells. The cells were thoroughly resuspended in the PPS, which was subsequently removed under the same centrifugation conditions as previously described. Following two cycles, an equivalent volume of PPS supplemented with 1 mM tryptophan was added and incubated in an anaerobic workstation for 24 h. Subsequently, the mixture was centrifuged at 8000 × *g* for 10 min, and the supernatant was filtered through a 0.22 μm filter membrane for subsequent detection.

### Determination of IA content in feces of mice and bacterial culture supernatant

The collected fecal samples were subjected to lyophilization under conditions of −107°C and 0.2 mbar. The freeze-dried fecal samples were precisely weighed to 50 mg, and subsequently augmented with 800 μL of a methanol aqueous solution (methanol: water = 1: 1). The samples underwent vortex mixing for 15 s, followed by tissue crushing at a frequency of 65 Hz, each cycle lasting 30 s (repeated 5 times). The homogenized samples were incubated at 4°C overnight. Following centrifugation at 16000 × *g* for 10 min, 500 μL of the supernatant was aspirated and concentrated under vacuum at 45°C for 2–4 h. The supernatant was resuspended in 200 μL of a methanol solution (methanol: water = 1: 9), centrifuged at 14000 × *g* for 10 min, and subsequently filtered through a 0.22 μm filter membrane.

A stock solution of IA with a concentration of 1,000 ppm was prepared by diluting a methanol-aqueous solution (methanol: water = 1: 9) to yield concentrations of 50 ppm, 25 ppm, 10 ppm, 5 ppm, 1 ppm, 500 ppb, 250 ppb, 125 ppb, 100 ppb, 50 ppb, 25 ppb, 12.5 ppb, 6.25 ppb, 3.125 ppb, and 1.5625 ppb. These varying concentrations of IA were employed to construct a standard curve correlating peak area with concentration. IA was quantified using ultra-performance liquid chromatography coupled with high-resolution mass spectrometry (UPLC-HRMS; Thermo Fisher Scientific, Waltham, MA, USA; C18 UPLC Columns). The column temperature was maintained at 36.5°C, while the automatic sampler temperature was set at 4°C. Each sample was analyzed in positive ion mode with mobile phases comprising (A) acetonitrile and (B) 0.1% formic acid in aqueous solution. The injection volume was 2 μL, and the flow rate was 300 μL/min. The gradient elution program settings are detailed in Table 1.

**Table 1:** Gradient elution program settings.

Time (min)	Percentage of mobile phase A (%)	Percentage of mobile phase B (%)
0–3	5	95
3–9	30	90
9–15	100	0
15–16.5	100	0
16.5–20	5	95

### High throughput sequencing of the gut microbiota

The FastDNA Spin Kit for Feces (MP Biomedicals, CAT.NO.6570200) was employed to extract DNA from the fecal samples of mice, adhering strictly to the manufacturer’s protocol. The polymerase chain reaction (PCR) was utilized to amplify the DNA within the V3–V4 region. Post-PCR, the agarose gel was excised for purification purposes, utilizing the DNA Gel/PCR Purification Miniprep Kit (BW-DC3511, Beiwo Meditech Co., Ltd., Hangzhou, China), in strict accordance with the manufacturer’s guidelines. The PCR primers (341F and 806R), sequencing platform (Illumina NovaSeq 6,000), quality control method of the raw data, and analysis platform [QIIME 2 (19)] were referred to Lu et al. (20). Conduct a differential genus analysis utilizing the integrated LEfSe (Linear Discriminant Analysis Effect Size) utility within OmicStudio (21), accompanied by visualization, and employing the subsequent filters: Kruskal-Wallis test threshold set at 0.05; Wilcoxon test threshold set at 0.05; LDA score threshold set at 3.

### Statistical analysis

The allergy symptom scores, diarrhea index, ELISA results, and IA content were subjected to statistical analysis using IBM SPSS Statistics 26 (SPSS Inc., Chicago, IL, United States). The assessment of significant differences between the groups was conducted through one-way analysis of variance (ANOVA) followed by Duncan’s multiple range test. All data are presented as mean values ± the standard error of the mean (SEM). A *p*-value of <0.05 was deemed indicative of a statistically significant difference between the groups, corresponding to a confidence interval exceeding 95%.

## Results

### Oral administration of CCFM1189 and CCFM1190 cannot alleviate food allergy symptoms in mice treated with ABX

**Figure 2:**
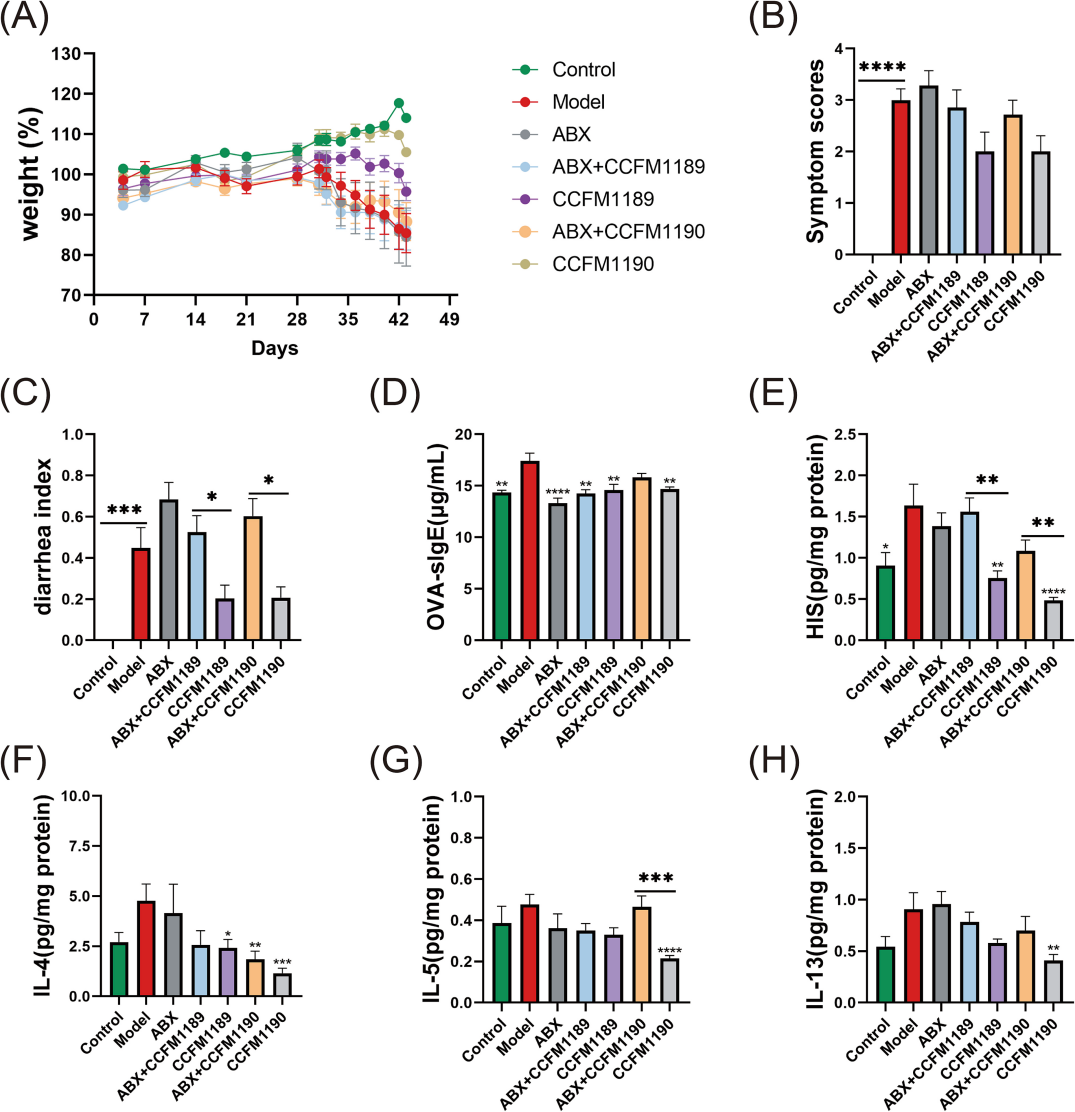
Alterations in allergy-related biomarkers and cytokine concentrations in both normal food allergic mice and food allergic mice subjected to ABX treatment, with the intervention of effective probiotic strains. **(A)** The weight (%) changes from day 0 to day 43. The symptom scores **(B)**, diarrheal indexes **(C)**, levels of serum OVA-sIgE **(D)**, levels of jejunal tissues’ HIS **(E)**, IL-4 **(F)**, IL-5 **(G)**, and IL-13 **(H)** of food allergic mice on day 43. The result represents mean ± SEM. The *p*-value were measured using one-way ANOVA with Duncan’s multiple range test (*p < 0.05, **p < 0.01, ***p < 0.001, ****p < 0.0001).

Initially, we performed exhaustive evaluations and statistical analyses on the food allergy-associated phenotypic indices across all experimental mice groups. In alignment with our prior research, CCFM1189 and CCFM1190 exhibited the outstanding capacity to alleviate weight loss, allergy scores, and diarrhea symptoms in normal food allergic mice (Figures 2A–C). Nevertheless, in contrast to these two groups, ABX treatment intensified the disease phenotypes in food-allergic mice, and the oral administration of CCFM1189 and CCFM1190 failed to significantly alleviate the symptoms in ABX-treated mice.

Subsequently, we determined the levels of serum OVA-sIgE across all mice groups. As shown in Figure 2D, administration of ABX resulted in a reduction of serum OVA-sIgE levels in mice when compared to the model group. In the case of CCFM1189 intervention, no significant difference was observed in the response to OVA-sIgE whether or not ABX was treated. Regarding the CCFM1190 group, it is exhibited a significant reduction in serum OVA-sIgE levels in mice (*p* < 0.01), whereas the ABX-treated CCFM1190 group did not demonstrate such a reduction. The results of jejunum HIS levels (a critical mediator in allergic responses; measured in supernatant) showed that effective bacterial strains have the potential to substantially mitigate the heightened HIS levels induced by food allergies; however, probiotic interventions in mice subjected to ABX treatment failed to diminish the HIS content within the jejunum (Figure 2E).

The levels of Th2 cytokines (IL-4, IL-5, and IL-13) in the jejunum supernatant of mice indicated that ABX treatment mitigated the inhibitory impact of the effective strains CCFM1189 and CCFM1190 on the Th2 immune response. In comparison to the model group, CCFM1189 and CCFM1190 notably diminished the IL-4 levels in the jejunum of mice, with the alleviating effect of the ABX-treated strain being less pronounced (Figure 2F). Regarding IL-5 and IL-13, CCFM1189 decreased the levels of these cytokines; however, this reduction was not statistically significant when compared to the ABX + CCFM1189 group; CCFM1190, in the absence of ABX treatment, significantly lowered the levels of IL-5 (Figure 2G) and IL-13 (Figure 2H; *p* < 0.01). Collectively, the ABX + CCFM1189 and ABX + CCFM1190 groups exhibited a lesser inhibitory effect on the Th2-type immune response in food allergic mice compared to the CCFM1189 and CCFM1190 groups.

**Figure 3:**
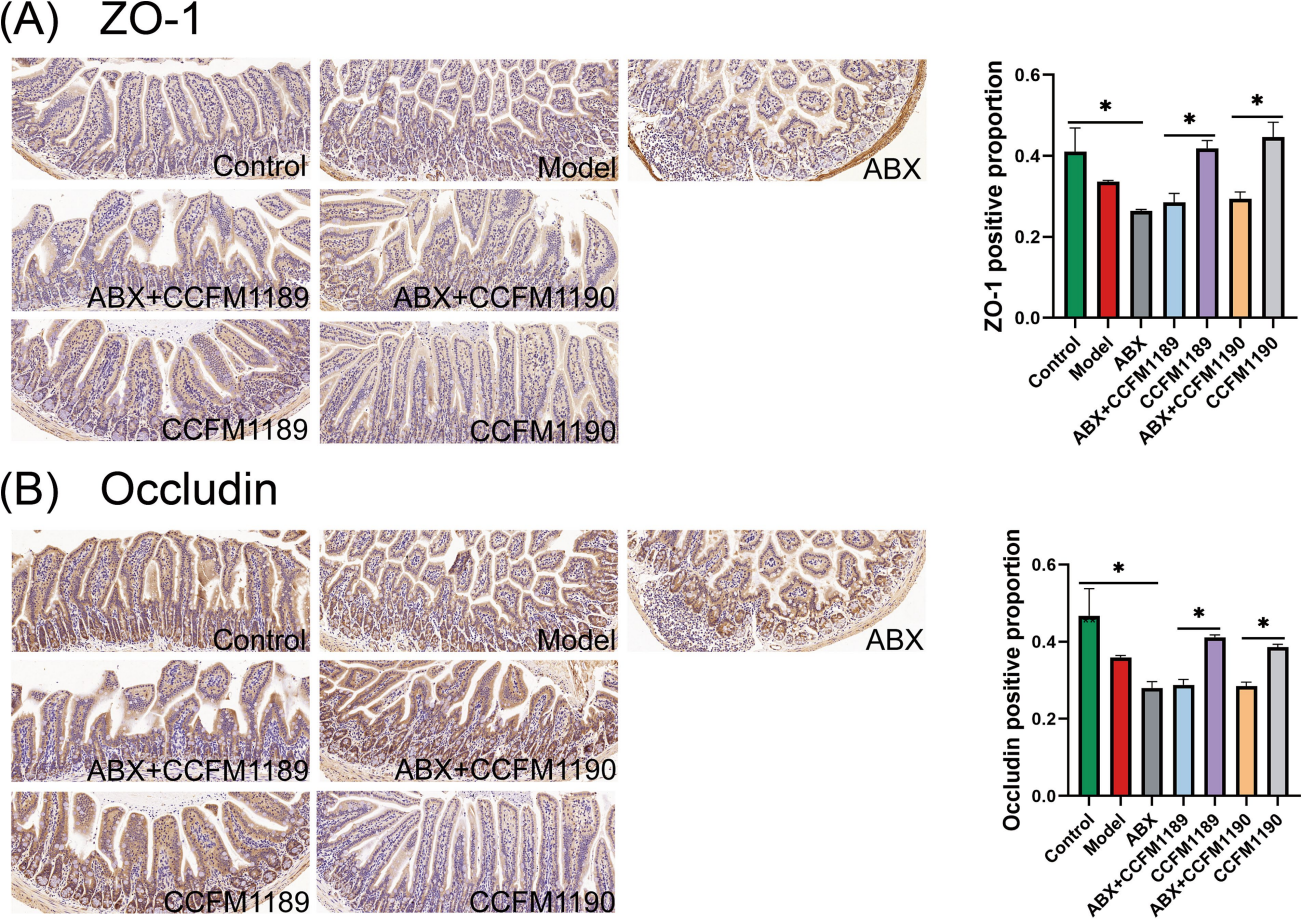
Alterations in intestinal barrier markers in both normal food allergic mice and food allergic mice subjected to ABX treatment, with the intervention of effective probiotic strains. **(A)** The pathological sections of the jejunum with immunohistochemistry (ZO-1, 20×) and the positive percentage of ZO-1. **(B)** The pathological sections of the jejunum with immunohistochemistry (Occludin, 20×) and the positive percentage of Occludin. The result represents mean ± SEM. The *p*-value were measured using one-way ANOVA with Duncan’s multiple range test (*p < 0.05, **p < 0.01, ***p < 0.001, ****p < 0.0001).

Moreover, the immunohistochemical analysis of tight junction proteins ZO-1 and Occludin in the jejunum of mice was conducted, with the proportion of positively stained regions being quantified (Figures 3A,B). The results indicated that ABX treatment led to a reduction in the expression levels of ZO-1 and Occludin in the jejunum of mice. The expression levels of ZO-1 and Occludin in the ABX + CCFM1189 and ABX + CCFM1190 groups were markedly lower compared to those in the CCFM1189 and CCFM1190 groups without ABX treatment (*p* < 0.05). After histopathological examination of the jejunum sections in mice, it is found that the small intestinal villi of the model group mice exhibited disorganization and abscission, while the lamina propria appeared disordered and lax. The intestinal villi of mice in the CCFM1189 and CCFM1190 groups were orderly arranged, with the lamina propria being intact and closely aligned, resembling the phenotype of the control group and thereby restoring the integrity of the intestinal barrier to a certain extent. In contrast, the ABX treatment groups (ABX, ABX + CCFM1189, and ABX + CCFM1190) displayed comparable phenotypes and elevated levels of inflammatory cell infiltration, akin to the model group.

### The effective strains themselves do not produce IA, but can augment the intestinal concentration of IA by modulating the gut microbiota

**Figure 4:**
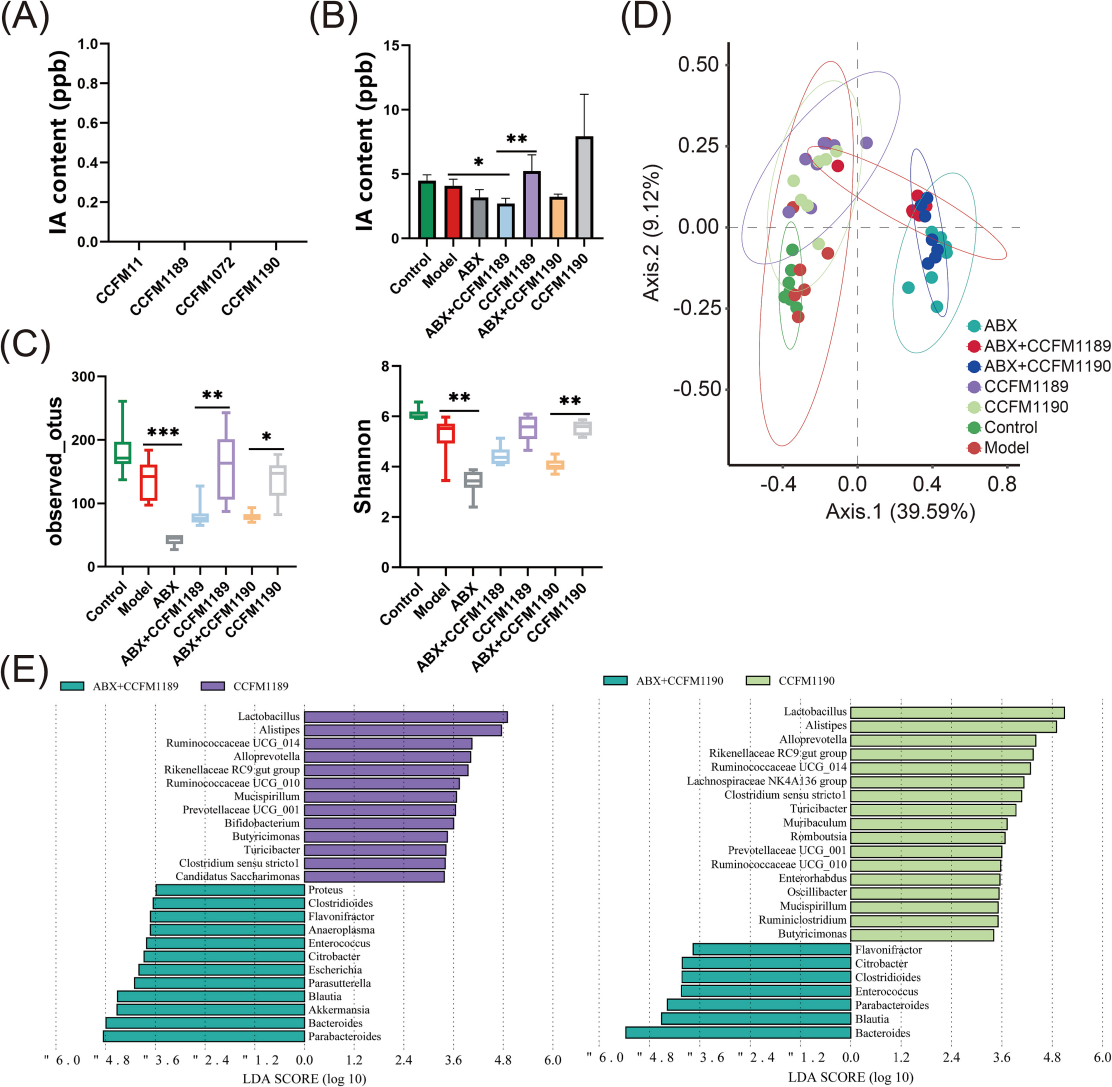
Alterations in intestinal IA levels and microbiota in both normal food allergic mice and food allergic mice subjected to ABX treatment, with the intervention of effective probiotic strains. **(A)** IA content *in vitro* fermentation. **(B)** The IA content in the feces of mice. **(C)** Changes in alpha diversity index. **(D)** Bray-Curtis principal coordinate analysis (PCoA) of beta diversity between the groups. **(E)** The differences in bacterial genera between the CCFM1189 group and ABX + CCFM1189 group, as well as between the CCFM1190 group and ABX + CCFM1190 group. The result represents mean ± SEM. The *p*-value were measured using one-way ANOVA with Duncan’s multiple range test (*p < 0.05, **p < 0.01, ***p < 0.001, ****p < 0.0001).

In our prior research endeavors, we have ascertained that the probiotic strains CCFM1189 and CCFM1090 significantly mitigate food allergy in ovalbumin-induced mice, with IA being identified as a crucial microbial-derived metabolite. To elucidate the mechanisms responsible for the variations in IA levels within the gastrointestinal tract under probiotic intervention, we initially utilized an *in vitro* fermentation methodology. By incorporating tryptophan into the culture medium, we assessed the capacity of CCFM1189 and CCFM1190 to metabolize tryptophan and yield IA (Figure 4A). The results indicated that none of these strains were capable of producing IA through tryptophan metabolism during *in vitro* fermentation. Subsequently, to verify whether CCFM1189 and CCFM1190 augment the IA content in mouse feces by influencing other symbiotic bacteria within the intestinal tract, the IA content of effective strain groups treated with ABX (ABX + CCFM1189, ABX + CCFM1190) was compared with those not treated with ABX (CCFM1189, CCFM1190). The results demonstrated that the IA content in the feces of ABX-treated mice was lower than that of both the control and model groups. The fecal IA content of the CCFM1189 and CCFM1190 groups without ABX treatment exhibited an increase compared to the ABX-treated groups (Figure 4B). The aforementioned findings imply that CCFM1189 and CCFM1190 could potentially mitigate food allergy symptoms by augmenting the IA concentration in the fecal matter of mice, with the elevated IA levels possibly attributable to the probiotic strains’ influence on the intestinal symbiotic microbiota of the mice.

**Figure 5:**
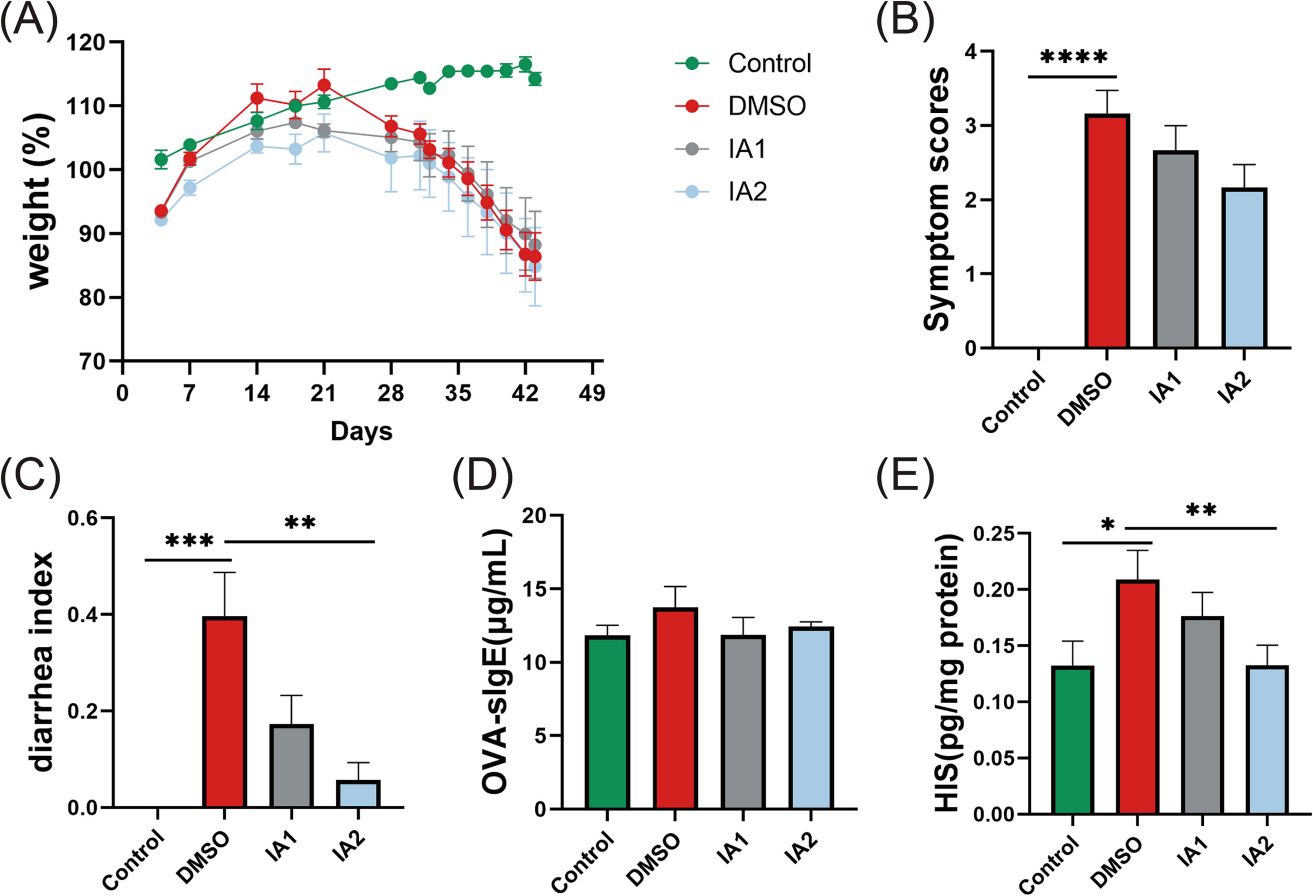
The impact of external IA intervention on food allergy related-indicators in mice. **(A)** The weight (%) changes from day 0 to day 43. **(B)** The symptom scores of food allergic mice on the day 43. **(C)** The diarrheal index of food allergic mice on the day 43. **(D)** The levels of serum OVA-sIgE of mice. **(E)** The levels of HIS in the supernatant of jejunal tissues. The result represents mean ± SEM. The *p*-value were measured using one-way ANOVA with Duncan’s multiple range test (*p < 0.05, **p < 0.01, ***p < 0.001, ****p < 0.0001).

Then, we conducted an analysis of the diversity and composition of the gut microbiota across various groups of mice. As depicted in Figure 4C, CCFM1189 and CCFM1190 exhibit the capability to reverse the reduction in microbial species diversity induced by food allergy models. Following treatment with ABX, a marked decrease in the diversity of the gut microbiota in mice was observed, and the intervention of probiotic strains failed to mitigate the dysbiosis induced by antibiotics (Figure 5A). The results pertaining to *β* diversity indicated that the distributions of ABX treatment groups (ABX, ABX + CCFM1189, and ABX + CCFM1190) was more concentrated, while were dispersed in the control, model, CCFM1189, and CCFM1190 groups (Figure 4D).

Moreover, an analysis was conducted to identify the differential bacterial genera within the gut microbiota of mice between the probiotic intervention group and the group receiving both ABX treatment and probiotic intervention. As shown in Figure 4E, it was observed that within the CCFM1189 group, the dominant bacterial species included *Lactobacillus*, *Alistipes*, *Ruminococcaceae UCG-014*, *Alloprevotella*, *Rikenellaceae RC9 gut group*, *Ruminococcaceae UCG-010*, *Mucispirillum*, *Prevotellaceae UCG-001*, *Bifidobacterium*, *Butyricimonas*, *Turicibacter*, *Clostridium sensu stricto 1*, and *Candidaatus saccharimonas*. In the CCFM1190 group and the ABX + CCFM1190 group, *Lactobacillus*, *Alistipes*, *Alloprevotella*, *Rikenellaceae RC9 gut group*, *Ruminococcaceae UCG-014*, *Lachnospiraceae NK4A136 group*, *Clostridium sensu stricto 1*, *Turicibacter*, *Muribaculum*, *Romboutsia*, *Prevotellaceae UCG-001*, *Ruminococcaceae UCG-010*, *Enterorhabdus*, *Oscillibacter*, *Mucispirillum*, *Rumiclostridium*, and *Butyricimonas* were found to be enriched within the CCFM1190 group. Among the bacterial genera enriched under the intervention of effective probiotic strains, *Clostridium*, *Prevotella*, *Bifidobacterium*, and *Lachnospira* have been identified as bacterial genera capable of producing tryptophan-indole derivatives (22). Consequently, we assert that the ingestion of the effective strains CCFM1189 and CCFM1190 can modulate the gut microbiota, promoting the proliferation of bacteria that produce elevated levels of IA and resulting in an increased concentration of IA within the gut.

### IA can directly relieve food allergic symptoms in mice

In order to further elucidate the mechanisms and underlying material basis through which probiotics effectively mitigate food allergies, we conducted direct interventions using low-dose and high-dose IA in murine models of food allergy. The results indicated that neither dosage of IA significantly influenced the weight loss observed in the mice (Figure 5A). However, in comparison to the DMSO group, the IA intervention exhibited remarkable efficacy in ameliorating the phenotypic manifestations (Figure 5B) and the diarrhea index (Figure 5C) in food allergy mice, with the high-dose IA demonstrating a more pronounced effect. Furthermore, neither dosage of IA produced a significant reduction in serum OVA-sIgE levels in food allergy mice (Figure 5D). Regarding the HIS levels in the jejunal tissue supernatant of the mice, both low-dose and high-dose IA were effective in decreasing HIS concentrations, with the high-dose IA showing a notably greater impact (Figure 5E).

**Figure 6:**
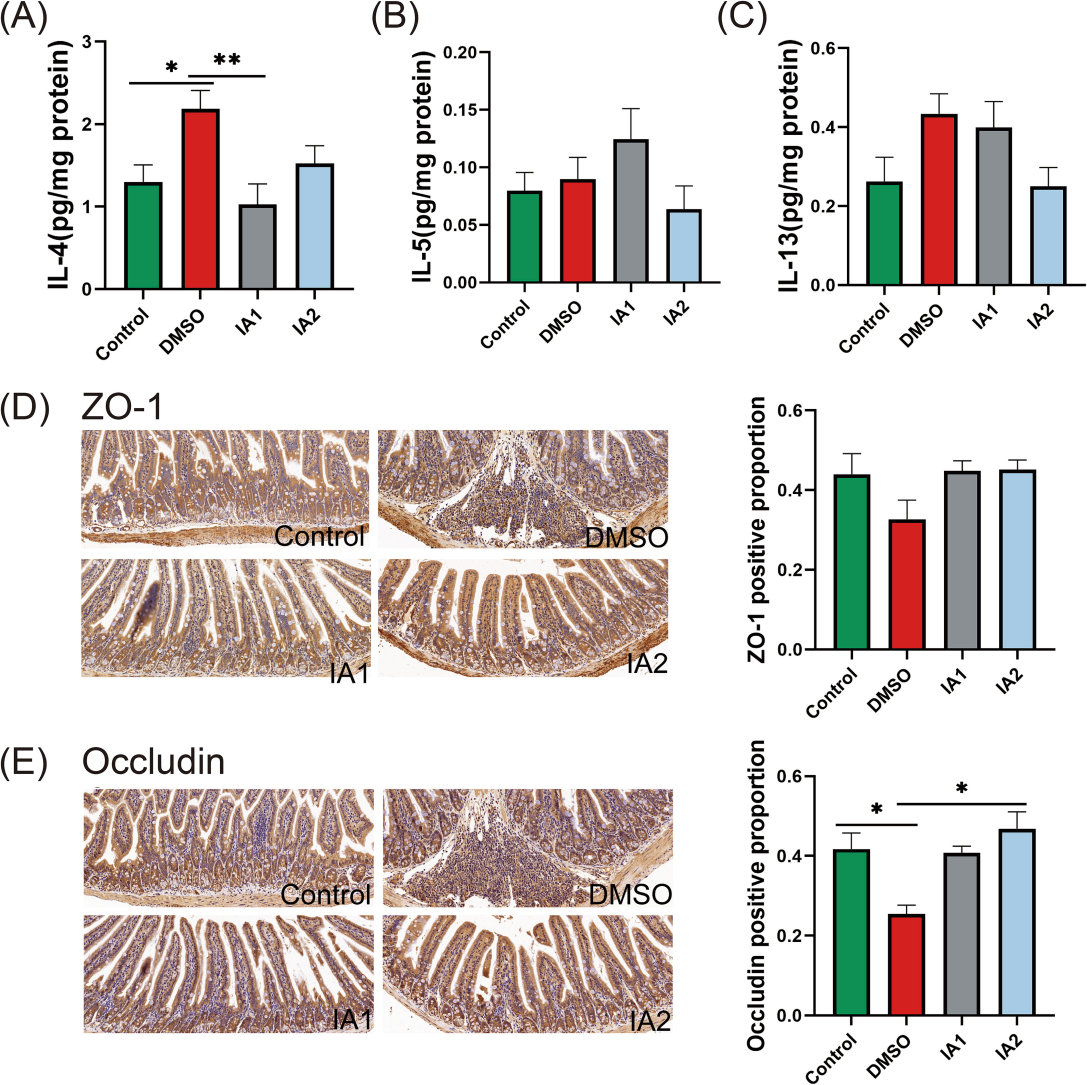
The impact of external IA intervention on cytokines and intestinal barrier in food allergic mice. **(A–C)** The levels of IL-4, IL-5, and IL-13 in the supernatant of jejunal tissues. **(D)** The pathological sections of the jejunum with immunohistochemistry (ZO-1, 20×) and the positive percentage of ZO-1. **(E)** The pathological sections of the jejunum with immunohistochemistry (Occludin, 20×) and the positive percentage of Occludin. The result represents mean ± SEM. The *p*-value were measured using one-way ANOVA with Duncan’s multiple range test (*p < 0.05, **p < 0.01, ***p < 0.001, ****p < 0.0001).

IA has the potential to inhibit the Th2-type immune response to a certain degree. In comparison to the DMSO group, low-dose IA was found to significantly reduce the levels of IL-4 in the jejunal supernatant of mice (Figure 6A). Regarding IL-5 (Figure 6B) and IL-13 (Figure 6C), high-dose IA exhibited a decreasing effect. Furthermore, IA was observed to enhance the positive expression of ZO-1 and Occludin immunohistochemical staining. In the DMSO group, the expression levels of ZO-1 (Figure 6D) and Occludin (Figure 6E) in the jejunum of mice were diminished; however, IA administration resulted in an elevation of these expression levels. Notably, there was no significant difference in the regulatory effects of IA on tight junction proteins between the two dosage levels. In conclusion, we have confirmed that IA serves as a crucial metabolite in the mitigation of food allergies and have elucidated the mechanisms of action of the probiotic strains CCFM1189 and CCFM1190.

## Discussion

Our prior investigation has revealed that the probiotic strains CCFM1189 and CCFM1190 mitigate OVA-induced food allergy in mice through the modulation of gut microbiota and the enhancement of IA levels in fecal specimens. Nevertheless, before that, it remained unclear whether CCFM1189 and CCFM1190 produce IA through their own fermentation processes or by regulating the intestinal symbiotic microbiota. Preceding research has documented those bacteria harboring the phenyllactate dehydrase gene cluster (fldAIBC) possess the capability to metabolize tryptophan, yielding IA and indole-3-proionic acid (23). With the exception of *Peptostreptocococcus*, neither *Lactobacillus* nor *Bifidobacterium* have been documented to harbor this gene cluster. The experimental outcomes of *in vitro* fermentation conducted in this study further corroborated that CCFM1189 and CCFM1190 are incapable of producing IA through the direct metabolism of tryptophan. In light of the aforementioned findings, it is plausible to infer that IA production is mediated by the regulation of gut microbiota.

Employing pseudo-germ-free mice as experimental subjects, we substantiated that these two probiotic strains capable of mitigating food allergies necessitate interaction with the gut’s endogenous microbiota. Zhang et al. (24) noted exacerbated allergic manifestations, including scratching, convulsions, and diminished activity, in mice subjected to antibiotic treatment, a result congruent with our observations. Nevertheless, the serum concentrations of OVA-sIgE in OVA-sensitized mice treated with ABX were marginally elevated compared to those in untreated mice, a discrepancy that does not correlate with the phenotypic symptom exacerbation. This incongruity may be attributable to certain antibiotics, such as tetracycline, potentially suppressing IgE production (25). Furthermore, we discovered that ABX treatment attenuated the inhibitory impacts of CCFM1189 and CCFM1190 on Th2 immune responses. This may be because antibiotic treatment perturbs the composition of gut microbiota and their metabolites, thereby modulating the Th1/Th2 balance (26). Zhang et al. (27) demonstrated that *Lactobacillus acidophilus* ST218 intervention could repress the elevation of Th2 cytokines IL-4 and IL-5 induced by airway inflammation in antibiotic-treated mice, a result consistent with our findings.

Alterations in tryptophan (Trp) metabolism have been documented to be intimately linked with a multitude of diseases (28). Multiple pro-inflammatory mediators and deleterious T cell activities are modulated by Trp and its metabolites, functioning as an adaptive regulatory mechanism to curtail excessive acute immune responses within tissues (29). Roughly 4–6% of tryptophan transport is conveyed to the large intestine, where it undergoes degradation by the gut microbiota. In the context of microbiome influence, tryptophan can be transformed into indoles and their derivatives, such as indole, tryptamine, indole-3-acetamide, indole-3-lactic acid, indole-3-propionic acid, indole-3-acetaldehyde, indole acetic acid, indole-3-aldehyde, and IA (30). These indole derivatives have been extensively identified to exhibit properties that suppress intestinal inflammation (17, 31), augment gut barrier function (32–34), boost host antioxidant capacity (35, 36), and regulate host immune responses (37, 38).

Our research has, for the first time, demonstrated that IA exerts a direct ameliorative effect on food allergy in mice. Despite the lack of an alleviative effect of IA intervention on weight loss in mice, it effectively mitigated allergic and diarrhea symptoms, as well as OVA-sIgE, HIS, and Th2-type cytokine levels in the jejunum, and enhanced the expression of tight junction proteins ZO-1 and Occludin, thereby alleviating intestinal injury induced by food allergy. As a metabolite of tryptophan, IA is capable of activating the AhR signaling pathway (39). The activation of this pathway subsequently inhibits Th2-type cytokines and antigen-specific antibodies generated by B cells, thereby modulating the Th2-mediated immune response (40). Tight junction proteins constitute a barrier at the apex of the adjacent epithelial cell membrane, thereby inhibiting paracellular molecular transport between cells. In conjunction with adherent connexins, these proteins assemble into an apical connexin complex, which plays a pivotal role in maintaining intestinal barrier integrity (41, 42). It has been reported that other small molecular tryptophan metabolites exert regulatory effects on the expression of intestinal tight junction proteins, thereby modulating intestinal barrier integrity (17, 23). For example, indole-3-propionic acid has been shown to upregulate mRNA encoding tight junction proteins and augment the expression of Claudin and Occludin (32). Additionally, indole-3-propionic acid can enhance the secretion of TFF3 and RELMβ by MUC2, MUC4, and goblet cells, thereby reinforcing the mucus barrier (34). Our findings suggest that IA enhances the expression of ZO-1 and Occludin in the jejunum tissue of food allergic mice. Consequently, these results imply that tryptophan indole metabolites possess a general capacity to augment intestinal barrier integrity.

## Conclusion

In summary, our investigation has elucidated the underlying mechanisms and material foundations through which probiotic strains *L. plantarum* CCFM1189 and *L. reuteri* CCFM1190 mitigate food allergies. Mice afflicted with food allergies and subjected to antibiotic treatment failed to exhibit symptom alleviation upon probiotic supplementation, thereby substantiating those efficacious strains must exert their effects via the gut microbiota. In addition, we also elucidate the pivotal role of IA in mitigating food allergies, explicate the underlying mechanism through which probiotics that do not produce IA manifest their effects, and endeavor to furnish scientific substantiation for prospective food allergy prophylactic and therapeutic approaches predicated on gut microbiota.

## Data Availability

The datasets presented in this study can be found in online repositories. The names of the repository/repositories and accession number(s) can be found at: https://www.ncbi.nlm.nih.gov/, PRJNA1182655.
